# Antiseptics and Disinfectants Used in Dental Surgery

**Published:** 1892-03

**Authors:** Saville H. Hayward


AMERICAN JOURNAL

—OF—

DENTAL SCIENCE.

VOL. XXV. BALTIMORE, MARCH, 1892. NO. 11.

ARTICLE I.

### ANTISEPTICS AND DISINFECTANTS USED IN DENTAL SURGERY.

BYSAVILLE H. HAYWARD.

A Paper read before the Students' Society, Dental Hospital of London.

*Coutinued from page 459.*

*Sulphuric acid* consisting of a solution of sulphurous acid gas in water possesses the properties both of a disinfectant and antiseptic. Freshly prepared by heating sulphuric acid with charcoal and dissolving the gas in water, it is a colorless liquid containing 5 per cent. by weight of sulphurous acid gas $SO_3$; having a great affinity for oxygen it readily decomposes organic bodies, and thus destroys low forms of living matter, arresting fermentation, and preventing the development of bacteria. As a devitalizer of bacteria, in experiments performed by N. de la Croix and confirmed by Koch, a high place was given it, a solution of 1:2000 being recorded as sufficient to kill them, while a concentration of 1 : 330 prevented the reproduction of their spores; on the other hand, germs have been found alive in a tube of bouillon impregnated with the gas after a lapse of fifteen days, the liquid having a strongly acid re-action. Its use in dental surgery is indicated in the cases of thrush or stomatitis, when it may be prescribed in the form of a lotion of one part to two or more of water combined with a little glycerince, or as a spray, which is a convenient method of application in ulcerative stomatitis. It may also be prescribed in the form of one of the alkaline sulphites or hyposulphites 3 gs. to ℥j of water. The sodii hyposulphis is specially indicated where the oral fluids are of an acid reaction, as in them it is decomposed, free sulphurous acid being evolved. As with peroxide of hydrogen and the preparation of chlorine, it is imperative that sulphurous acid and solutions of the hyposulphites should be used in a fresh state, since by the absorption of oxygen sulphuric acid is gradually formed.

In the second division of my subject it will be well, perhaps, to group together the coal car derivatives and the iodine compounds, and to speak of these first. Among the former we shall find of chief importance carbolic and salicylic acids and resorcin.

Pure carbolic acid, phenol, or phenilic acid C6 H5 OH is an alcohol obtained by fractional distillation from that part of coal tar, boiling between 180 deg. and 190 deg. C. Purified, it appears as a colorless crystalline body, but slightly soluble in water (1 in 14), but readily so in rectified spirit of wine, ether, glycerine, fixed and volatile oils, and becoming permanently liquified by the addition of ten per cent. of water. Among antiseptics it is perhaps the most widely used, being a most powerful germicide and disinfectant, a solution of 1 : 500 prevents the development of bacteria of the human mouth, and of 1 : 200 prevents the development of spores, and of 1 : 20 completely destroys them. Unlike chlorine, it is not a chemical disinfectant having no power of combining with and destroying mephitic gases. In its power of penetrating and sterilizing carious dentine, it is placed by Miller as inferior to bichloride of mercury and trichloride of iodine, and the results of his experiments in this direction, would seem to show that the acid requires to be much longer applied to the cavity to be disinfected than is usually the case, and that the mere swabbing out of the excavation previous to filling, is practically useless. The undiluted acid is strongly escharotic, coagulating albumen, and therefore contra-indicated as an antiseptic application to exposed pulps in the process of "capping," and should not even be used in a cavity where but a thin layer of dentine is left covering the pulp, or used as a dressing in the concentrated form where it is likely to come in contact with albuminous liquids. A theory started by Dr. Harlan, of the United States, seems to have gained ground in that country to the effect that its use is inadmissable in the treatment of root canals and pulpless teeth, since it coagulates the organic contents and does not penetrate the dentinal tubues, this statement hardly tallies with the result of Miller's experiments given above, and the objection seems to border on the fanciful, but its effects, except in a diluted form on albuminous substances, should be borne in mind in the treatment of root canals connected with abscess sacs and which have fine apical foramina, else the latter may become blocked, preventing free drainage. For permanent antiseptic dressings, it does not by any means form a suitable agent since it is readiy absorbed by the tissues, leaving no trace of its presence after the lapse of a few months. When chemically equivalent parts of strong carbolic and sulphuric acids are mixed, aseptol or sozolic acid, a viscous reddish liquid results, neither possessing the odor of phenol nor any corrosive or irritant properties; it was brought into notice by Dr. Huppe on the Continent, whose experiments led him to place it as an antiseptic with phenol and perchloride of mercury. Freely soluble in water a 10 per cent. solution was found to kill the spores of the anthrax bacillus in thirty minutes, a 5 per cent. solution of carbolic acid requiring twenty-four hours to produce the same effect, while a 3 or 5 per cent. solution was found to be a true disinfectant of micro-organisms free from spores; although soluble in alcohol and glycerine, solution in these media showed a non-antiseptic action; a great point in its favor is that it is hardly at all toxic in its nature, and a 10 per cent. solution; even, has no caustic action when applied to the skin. A 3 per cent. solution is recommended in pyorrhœa alveolaris to be injected between the gums and teeth every three days for a fortnight; by this means the flow of pus is said to be arrested, the gingival margin restored, and all tenderness removed. Carbolic acid is freely soluble in the caustic alkaline solutions; a mixture of 8 parts of phenol with 4 of caustic soda and 100 of water forms the liq. sodii carbolatis or phenol sodique, which, properly diluted, makes a most useful application where the combined antiseptic and disinfectant properties of the phenol and the alkaline re-action of the soda are desired. When carbolic acid is used as a mouth wash, it should be prescribed in this form, or with some substance neutralizing its causticity, such as glycerine.

*Salicylic Acid* is naturally present in certain essential oils, but is commercially produced by the action of carbonic acid gas on a mixture of carbolic acid and caustic soda, is possessed of great anti-fermentative and anti-putrefactive properties, experiments carried out by Goddefray showing it to be three times more powerful than carbolic acid as a preventive of fermentation. Both it and its compounds would appear to have a decided action on bacteria, prohibiting the growth of leptothrix, ciliated infusoria, and entirely preventing the development of anthrax bacilli, in a solution of the strength of 1 in 1,500. It possesses the advantage of having no smell, being comparatively harmless if taken internally, and of producing but little local irritation. It is but slightly soluble in cold water—1 in 700 or 800; but a 4 per cent. solution can be obtained by the addition of rather more than an equal quantity of borax; diluted to half this strength it forms a useful antiseptic mouth wash, correcting any fœtor of the breath arising from an unhealthy condition of the mouth. Its solution has a decidedly acid re-action, and its use in the mouth has been objected to by many who are of the opinion that it decalcifies the teeth; Miller says he has seen it used for years without any evil results, and advises its employment in a strength of 1:200 and 1:300, in which case he finds it capable of devitalizing a cultivation of ferment bacteria of the mouth in half a minute. Heated with camphor in the proportion of 84 parts to 65, salicylic acid forms on cooling a crystalline mass, salicylate of camphor or salicylated camphor, which, on being rubbed down, becomes of an unctuous consistence; it is found useful as an application in inflammatory conditions of the mucous membrane of the mouth.

Of the three isomeric compounds, derivatives of benzene, resulting from the replacement of two atoms of H by two of O H, viz.: resorcin, hydroquinone and pyrocatechin, all of which possess strong antiseptic properties, resorcin is the only one which has received much attention. It is produced by passing benzene vapor into hot sulphuric acid and heating the product with excess of soda; it occurs in white crystalline plates resembling benzoic acid and is soluble in less than two parts of water, in this strength it has a somewhat caustic action on the skin, but a 5 per cent. solution is not found irritating either to the skin or mucous membrane, and is capable of destroying the vitality of low organisms. It is recommended as an application in the treatment of mucous patches of the cheeks and gums, these being quickly cured by painting a 5 to 10 per cent. solution on the previously dried mucous membrane. As an antiseptic and disinfecting mouth wash, a weaker strength is advisable, a 2 or 3 per cent., otherwise an unpleasant tingling of the lips may be produced. Miller reports the power of resorcin in sterilizing carious cavities as very inferior to that of carbolic acid.

Though possessed of powerful antiseptic properties *iodine* is most generally employed in the form of one of its compounds, iodoform, iodol, aristol, and the trichloride of iodine.

Iodoform or tri-iodomethane, analogous in constitution to chloroform is prepared by the action of iodine on a hot solution of carbonate of sodium in dilute school, and occurs either in a minute crystalline, form or as an impalpable precipitate containing 29-30 of its weight of iodine, and possessing well marked antiseptic as well as slight sedative powers. It is insoluble in water, but dissolves in the proportion of 1:80 of spirit, 1:10 of ether, 1:12 of chloroform, and 1:14 of oil of eucalyptus. Heat decomposes it, liberating iodine and rancid oil having the same effect, it has been suggested that the good effects obtained when it is applied to an ulcerated surface may be due to decomposition taking place with the evolution of free iodine. Although *destroying* organisms less readily than carbolic acid, according to Miller, it is ten times as powerful in preventing their development, and it would appear to have a much more marked effect that it is as a disinfectant and deodoriser. A remarkable case illustrating this is given in the *Lancet*, of March, 1887, in which it is recorded that a "poisoned hand, riddled with abscesses," although kept in a carbolic bath for four hours, and then syringed and dressed with carbolic solution, 1:40 was in twelve hours as offensive as ever; but dressed with a 20 per cent. ointment of iodoform it was, after the lapse of the same time, perfectly sweet and remained so under that treatment until healed.

Iodoform has proved of value in the treatment of alveolar abscess and the dressing of root canals in a septic condition; for these purposes, Mr. Underwood recommends it to be combined with oil of eucalyptus; he records the successful treatment of several cases of a chronic nature, and says he has been able to retain roots by this means, whose preservation seemed otherwise hopeless. As a temporary dressing for root canals it is, perhaps, best applied in the form of iodoform wool containing 10 per cent. of iodoform (or if a wool more strongly medicated be desired the gossypium iodoformi of the Throat Hospital, containing 59 per cent.), this should be dipped in oil of eucalyptus being introduced into the canal; for permanent dressings the wool may be first dipped in melted iodoform wax or in a saturated solution of iodoform in collodion, 1:10.

Iodoform seems to be particularly fatal to the virus of syphilis, and in form of pastilles, 1 gr. to 18 gr. of glyco-gelatine is found useful in the treatment of syphilitic affections of the mouth and tongue.

When applied externally, as in the treatment of alveolar abscess bursting through the skin, the iodoformi pulvis should be used, as it has less tendency to clot than the precipitated preparation, and its extremely unpleasant and persistent smell should be masked, perfectly covered it cannot be by the admixture of coumaron, 1:50.

On account of its tonic properties and its disagreeable smell, several compounds possessed of like antiseptic properties, but odorless or non-poisonous bodies have been introduced, viz.: iodol, aristol, and sozoidol.

*Iodol.*—A micro-crystalline brownish powder prepared by precipitating pyrrhol (a derivative of animal oil) with iodo-iodide of potassium contains about 9-10ths of its weight of iodine. It is not soluble in water but in 34 parts of glycerine in 6 of alcohol, and freely in ether or collodion. It is non-irritant and non-toxic, and has no unpleasant smell. Depending, doubtless, like iodoform, for its antiseptic powers on the iodine in its composition, it can be employed whenever the use of the latter is indicated; the glycerine solution will be found specially useful for temporary dressings of pulpless teeth in a septic condition, and it has been recommended mixed with twice its weight of oxide of zinc to be made into a paste with vaseline, and used as a permanent filling for pulp chambers.

*Aristol* was first introduced about the beginning of last year, and has been used in general surgery to a considerable extent on the Continent. It is a reddish brown amorphous powder thrown down as a precipitate on pouring a solution of iodine in iodide of potassium into an alkaline solution of thymol. Its chemical constitution is not quite certain, but it would seem likely that a hydrogen atom of the hydroxyl group in thymol is replaced by iodine, forming an iodoxyl of thymol ($C_{10} H_{13} IO$). It contains 14.8 per cent. of iodine and though in this respect vastly inferior to iodoform, it has the great advantages of being very nearly tasteless, and odorless, and non-poisonous, while possessing marked antiseptic and antiparasitic action. It is insoluble in water, alcohol, and glycerine, but freely soluble in ether and the fatty oils. It is decomposed by heat with escape of free iodine, and when applied externally should be preserved from the light which has the same effect upon it. I have found it useful in the treatment of pulpless teeth, carrying it into the pulp canal in the form of powder on a Donaldson bristle, afterwards dressing the tooth with cotton wool dipped in oil of eucalyptus, upon which the aristol is sprinkled.

*The Sodium Salt of Sozoidal* (Di-iodophenol sulphonic acid) does not seem to have been used to any extent in this country, though I believe it has been in America. Its composition would indicate it as likely to be serviceable since the acid radicle contains 54 per cent. of iodine, 7 per cent. of sulphur, and 20 per cent. of phenol. It occurs in odorless shining circular crystals, soluble in 14 parts of water, and so diluted, does not appear to be of an irritating nature.

*Trichloride of Iodine* has been lately very favorably spoken of by Dr. Miller, who says it was recommended as an antiseptic in 1887, by Riedal and Sangenbach. It is, he says, a brick-red powder with an irritating smell of chlorine, the aqueous solution of which has an acid reaction. It is claimed for it that a 0·1 to 0·5 per cent. solution is equal in antiseptic power to a 0·5 to 1·0 per cent. solution of perchloride of mercury, while as a sterilizer of carious dentine, Miller thinks that a 5 per cent. solution is "one of the most active agents at our demand." A solution of this strength has an exhaustive action, though but slight when compared with pure phenol, and "in aqueous solutions having equal effects on spores of anthrax bacilli, sublimate is 5 to 6 times, and creosote 7 to 8 times, as poisonous as the trichloride of iodine."

*Perchloride of Mercury* has been regarded, and perhaps very properly, as the king of antiseptics, and would be doubtless still more extensively used than it is were it not for its extremely poisonous properties. According to Koch, a solution of one in a million retarded, and 1:300,000 completely prevented the development of anthrax bacillus, while a strength of 1:100,000, Miller says, is sufficient to prevent the development of mouth bacteria, and 1:1,000 destroys their spores, provided the latter are exposed to its action for a sufficient length of time. When a solution of sublimate is brought in contact with albuminous bodies, a heavy precipitate of albuminate of mercury is found which is not antiseptic, and in which organisms can grow and thrive. Professor Laplace, working in Koch's laboratory, discovered that if the solution be acidulated with HCl or tartaric acid there was no precipitate, while the perchloride suffered no diminution, but rather gained in antiseptic power. He recommends 1 part of mercury with 5 parts of tartaric acid to 1,000 parts of water. This result is, I should think, owing to the formation in the presence of albuminous substances, of a soluble acid albumen, since the solution responds to the chemical tests for $HgCl_2$, but it may be due to the known power of tartaric acid in preventing precipitates under certain conditions. For sterilizing carious cavities, Miller gives corrosive sublimate a very high place, but second to terchloride of iodine, and he recommends a 5 per cent. solution for this purpose; the best and most convenient vehicle being, I think, glycerine, in which it is soluble in the proportion of 2:3 by weight; he recommends its solution of 1:2,000, especially if combined with other antiseptics and disinfectants as an excellent antiseptic mouth wash, but its unpleasant taste and the peculiar susceptibility of some persons to its action would greatly control its use, except as an occasional remedy. Quoting Weitzel, he says the latter is enthusiastic in his praises of sublimate in the treatment of septic and putrid conditions of the mouth, and that he found "syringing the alveoli with a solution of 1:1,000 followed by the injection of 6 to 8 drops of a 2 per cent. solution in the septic parts, a most sovereign remedy in cases of septic alveolitis."

*Chloride of zinc* results from the direct action of HCl on the metal as a white, very deliquescent caustic substance, having strong and peculiar powers as an antiseptic, disinfectant, and deodorizer. It is freely soluble in water, alcohol and ether. As a preventor of the development of bacteria it is only half the strength of carbolic acid, but it has this remarkable effect, that if its solution is painted on the surface of a wound, the tissues of the latter are rendered incapable of putrefaction for a considerable time, two or three days, notwithstanding exposure to septic influences. It has, consequently, been recommended as an antiseptic application after operations in the mouth for tumors and affections of the tongue. A 5 per cent. solution is very useful as a deodorizing and cleansing mouth wash in cases of purulent, fœtid discharge within the oral cavity, and for injecting into the sinuses connected with necrosed bone; while a solution of a weaker strength may be injected with advantage in cases of empyema of the antrum. It not infrequently occurs that the anterior root canals of lower molars are exceedingly small and tortuous in their course, so that it may be extremely difficult, if not impossible, to entirely remove every part of the necrosed pulp. Under such circumstances, after the removal of as much as possible, the application of deliquesced zinc chloride, by means of a Donaldson's bristle, seems to give good results. It would seem in saturated solution to be about equal in strength with carbolic acid in sterilizing carious dentine, but its extreme escharotic action, which on account of its great power of penetrating tissue, would be dangerous if used in close proximity to a living pulp, and the great pain sometimes caused by its application almost preclude its use in this connection.

*Boracic* or *boric acid*, first introduced in Sweden as a preserver or food, has taken a prominent place as an antiseptic and disinfectant in surgery. It occurs in the form of white odorless crystals, having an unctuous feeling to the touch, and is usually obtained by the action of sulphuric acid on borax. A solution of 1:800 is stated by Koch to prevent the development of anthrax bacilli, while a strength of 1:130 is found to prevent the development of bacteria in general; though for complete sterilization of a liquid containing a ferment bacterium of the mouth, Miller found a concentration of 1:50 necessary for a period of more than fifteen minutes. It is soluble in 26 parts of water and 5 of glycerine. When 92 parts of the latter are heated with 42 parts of boric acid, a tough deliquescent mass, freely soluble in water and alcohol, is produced. Boroglyceride, which in solution of 5 or 10 per cent. strength, form a valuable application as injections or mouth washes, where a non-stimulating antiseptic is required.

Closely allied to the coal tar derivatives is *Creosote*, a product of the distillation of wood tar, the antizymotic powers of which are very similar to those of carbolic acid, destroying low vegetable organisms and the fermentation they cause. Unlike it, however, though soluble in alcohol, ether and oils, it is insoluble in glycerine; and only in 1,000 parts of water; in this latter strength it was found by Bucholtz to destroy bacteria. Its use, like that of carbolic acid, is controlled by its power of coagulating albumen, and further by its extremely unpleasant biting taste, and the salivation it produces.

*Among the Essential Oils*, it will, I think, only be necessary to speak of those of cinnamon, cloves, peppermint, eucalyptus, thyme and wintergreen. These all possess the power, on exposure, of absorbing oxygen, having an ozonizing influence on the surrounding atmosphere, and their antiseptic powers are increased by the amount of oxygen absorbed. In common with other essential oils, they consist of a mixture of liquid hydrocarbons, elæoptins, and oxidized hydrocarbons, stearoptins, the latter being either in the form of a solid camphor like body crystallizing out from the oil; or, being in the liquid form, are of a higher specific gravity than the elæoptins, and only distil over at a higher temperature than them. It will hardly be necessary to discuss at length the oils of wintergreen, cinnamon, cloves and peppermint. *Oil of Cloves*, which Koch found to *retard* the development of anthrax bacilli in a strength of 1:5000, probably owes its slight antiseptic powers to a salicylic compound it contains. *Oil of wintergreen* consists almost entirely of salicylate of methyl, and has a mild antiseptic action devitalizing ferment bacteria of the mouth, in a concentration of 1:300, in rather over fifteen minutes; these are both inferior in strength to the oil of peppermint, which Miller states he has found possessed of considerable action, a solution of 1:300 being capable of sterilizing a liquid containing a culture of a ferment bacterium of the mouth in from five to ten minutes.

*Oil of Eucalyptus*, obtained from the leaves of the blue gum tree, indigenous in Australia, has a very extensive use in antiseptic surgery. It consists of a mixture of a simple hydrocarbon and an oxidized product, the quality of the latter and its antiseptic activity increasing with age; the more volatile portion of the oil, consisting of the unoxidized hydrocarbon, is a mixture of terpene and cymene. Oil of eucalyptus in a dilution of 1:116 kills developed bacteria, prevents their development in 1:600, and the development of their spores in a strength of 1:300. Mr. Underwood recommends it strongly in the form of an injection in cases of alveolar abscess of a chronic form, and its persistence and diffusibility render it very useful in the treatment of pulpless teeth. Together with the other essential oils, Miller regards it as but a very poor agent in the sterilization of carious dentine; but I think his experiments in this direction are hardly conclusive since he ascribes their poor action to their insolubility in the juices of the dentine, and it should be a rule in treating dentine with essential oils to first dehydrate it with ethylic alcohol, dissipating the spirit, if the tooth be not too sensitive with the hot air syringe.

*Thymol.*—A stearoptine contained in oil of thyme, appears as large transparent rhomboidal crystals, having an aromatic taste, a thyme like odor, and having a caustic action on the skin. It is soluble in 800 parts of water, in 200 of glycerine, and freely in alcohol, ether and caustic alkaline solutions. It is a powerful antiseptic, 1:100 arresting fermentation, and 1:1500 rendering sterile a liquid containing a cultivation of a ferment bacterium of the mouth. Rubbad with equal parts of camphor, or phenol, an oily liquid results, convenient as an application on cotton wool in the treatment of pulpless teeth or teeth with necrosed pulps; and thymol itself in small pieces forms a very good application to leave in the pulp chamber of a tooth upon which it is deemed advisable to perform rhizodontrophy.

I have to thank you, Mr. President and gentlemen, for your kind attention to my paper, on a subject which I regret I have found myself only able to treat in so tame and unworthy a manner, but I trust that it may be of some service, if only by its faults, in starting a discussion that may be both interesting and useful.—*Dental Record.*

